# Chromodomain protein Tcd1 is required for macronuclear genome rearrangement and repair in *Tetrahymena*

**DOI:** 10.1038/srep10243

**Published:** 2015-05-19

**Authors:** Jing Xu, Yajing Yuan, Aihua Liang, Wei Wang

**Affiliations:** 1 College of Life Science, Shanxi University, Taiyuan, 030006, China; 2 Key Laboratory of Chemical Biology and Molecular Engineering of Ministry of Education, Institute of Biotechnology, Shanxi University, Taiyuan, 030006, China

## Abstract

The survival of an organism’s progeny depends on the maintenance of its genome. Programmed DNA rearrangement and repair in *Tetrahymena* occur during the differentiation of the developing somatic macronuclear genome from the germ line micronuclear genome. *Tetrahymena* chromodomain protein (Tcd1) exhibited dynamic localization from the parental to the developing macronuclei. In the developing macronuclei, Tcd1 colocalized with Pdd1 and H3K9me3. Furthermore, Tcd1 colocalized with Pdd1 in the conjusome and “donut structure” of DNA elimination heterochromatin region. During the growth and conjugation stages, *TCD1* knockout cells appeared normal and similar to wild-type strains. In addition, these knockout cells proceeded to the 2MAC-1MIC stage. However, the progeny of the *TCD1* knockout cells did not grow upon return to SPP medium and eventually died. The deletion of the internal elimination sequence R element was partially disrupted in the developing new macronuclei. Gamma H2A staining showed that Tcd1 loss induced the accumulation of DNA double-strand breaks and the failure of genome repair. These results suggest that the chromodomain protein Tcd1 is required for the rearrangement and repair of new macronuclear genome in *Tetrahymena*.

The genome integrity of different organisms is continuously challenged by multiple internal signals and external factors. In general, programmed DNA rearrangement and repair occur on a genome-wide scale in ciliated protozoa. Some ciliate cells excise thousands of DNA elements from the genome, break the chromosomes into hundreds of small units, and extensively remodel the genome during sexual development^1^. Therefore, the organisms have evolved specific molecular mechanisms to precisely and efficiently repair the broken ends generated by these events^2^. Among these protozoans, *Tetrahymena* is an important and useful modern organism for exploring the molecular mechanism underlying genome rearrangement and repair. *Tetrahymena* has two structurally and functionally different nuclei during the vegetative growing stage^3^. The macronucleus (MAC) is transcriptionally active and provides the somatic functions of cells, whereas the micronucleus (MIC) is transcriptionally silent during the vegetative growth^4^. The germline MIC and the somatic MAC are derived from the same zygotic nucleus during *Tetrahymena* sexual conjugation stage. Two types of genome rearrangement occur during new MAC formation. The first genome rearrangement is the deletion of internal eliminated sequences (IESs), which is accompanied by the ligation of the flanking macronucleus-destined sequences^5^. The other rearrangement involves chromosomal breakage followed by the deletions of breakage eliminated sequences and the addition of telomeres. Programmed genome rearrangements eliminate ~15% of the genome during conjugation^6^. Subsequently, the developing new macronuclear chromosomes endoduplicate to ~45 times of the haploid genome. In the absence of consensus DNA sequences in the process, IESs are targeted for elimination by an epigenetically regulated RNAi-related mechanism^7^. During early conjugation stage, the micronuclear genome transcribes and produces double-stranded RNAs that are diced into 28 to 30 nucleotide scnRNA by the Dicer-like protein Dcl1^8^,^9^. The scnRNAs then form complexes with the Argonaute protein Twi1^10^. The RNA helicase Ema1 facilitates interaction between the Twi1–scnRNA complex and the nascent ncRNAs in the parental MAC^11^. In the new MACs, the IES-specific scnRNA-Twi1 complexes target homologous sequences for chromatin modifications via the accumulation of H3K27me3 and H3K9me3. Furthermore, the chromodomain protein Pdd1 binds both H3K9me3 and H3K27me3, pdd3 preferentially recognizes H3K9me3^12^. The domesticated pi*ggyBac* transposase Tpb2p is responsible for the DNA cleavage during programmed DNA deletion^13^. The nonhomologous end-joining (NHEJ) core component TKu80 protein serves an end-protective role after DNA cleavage^14^. In the final step, Ligase IV and Xrcc4p are required for the covalent joining of the two broken ends^2^. A higher order heterochromatin structure is essential during this process to achieve effective genome rearrangement and NHEJ genome repair.

A chromatin organization modifier (chromo) domain is a 40 to 50 amino acid region that was originally identified as a conserved sequence motif between polycomb proteins and heterochromatin protein-1 (HP1) from *Drosophila melanogaster*^15^,^16^. Chromodomain-containing proteins can interact with diverse targets, including histones, DNA, and RNA^17^. The three “caging” aromatic residues of a chromodomain are necessary for binding trimethylated H3K9 and H3K27^18^. The tandem chromodomains of human CHD1 act cooperatively to specifically bind methylated H3K4^19^. The histone acetyltransferase MOF specifically binds through its chromodomain to roX2 RNA^20^. Chromodomains at the integrase C termini of chromoviruses direct the integration of retrotransposons to heterochromatin^21^. Recent studies have revealed that HP1 promotes early DNA damage response events, which affects the efficiency of DNA repair in mammals^22^. These reports suggest that chromodomains have evolved from a common ancestral fold to fulfill various functions in different molecular contexts and execute multiple functions depending on the developmental context^17^,^23^.

In the present study, we characterized a novel chromodomain protein Tcd1 (*Tetrahymena* Chromodomain protein 1). Tcd1 colocalized with H3K9me3 and Pdd1 in the developing macronuclei, specifically in the conjusome body and the IES elimination region. The progeny of the *TCD1* germline knockout cells did not grow upon returning to SPP medium and eventually died. The deletion of the internal elimination sequence R element was disrupted in the newly developing new macronuclei and γ-H2A.X foci were maintained throughout the late conjugation stage in *TCD1* knockout strains. These results suggest that Tcd1 plays roles in genome rearrangement and repair in *Tetrahymena.*

## Results

### Identification of *TCD1*

**Figure 1:**
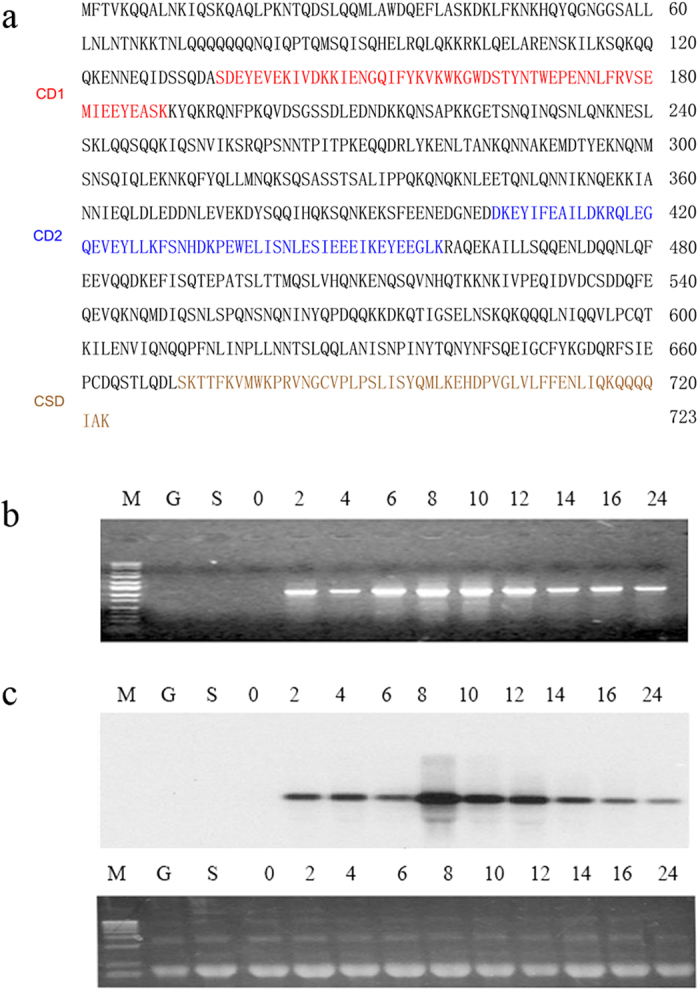
Characterization of *TCD1* (a) *TCD1*-Derived Protein Sequence. CD1 (chromodomain 1) and CD2 (chromodomain 2) are the regions that are similar to the canonical chromodomain. Both sequences are underlined. CSD is the chromoshadow domain. (**b**) Expression pattern of the *TCD1* gene. Total RNA from cells undergoing vegetative growth (G), starvation (S), and conjugation (at 0 h, 2 h, 4 h, 6 h, 8 h, 10 h, 12 h, 14 h, 16 h, and 24 h) after mixing were used. The obtained fragments were amplified by RT-PCR with primers for the *TCD1* coding sequences. (**c**) Northern blot probed with the *TCD1* coding sequence. The lower panel shows the loading control of rRNA stained with ethidium bromide before blotting. The samples derive from the same experiment and that gels have been run under the same experimental conditions and were processed in parallel.

To understand the function of chromodomain-containing proteins in genome organization, we identified 14 chromodomain-containing genes (including *PDD1, PDD3*, and *HHP1*) in the *Tetrahymena* genome database (http://www.ciliate.org) via a BLAST search with the conserved chromodomain sequence. Among these genes, TTHERM_01337400 (henceforth named *TCD1*) was not expressed in growing and starved cells; however, the mRNA expression of this gene was initiated at 2 h of mating and significantly increased between 8 h and 12 h of mating (Figs. 1b,1c). RT-PCR, 5′-RACE, and 3′-RACE were performed to obtain the complete mRNA sequence of *TCD1*. The open reading frame, 5′ UTR, and 3′ UTR of this gene comprise 2172, 37, and 165 bases, respectively. A comparison of cDNA and genomic DNA indicated that the cDNA contained four exons and encoded a putative 723 amino acid protein. Primary sequence analysis showed that Tcd1 has two chromodomains and one chromoshadow domain (labeled CD1, CD2, and CSD in Fig. 1a). CD1 resembles the canonical chromodomains in its primary sequence, whereas CD2 shares 38% identity with CD1. The expression pattern of *TCD1* is consistent with the microarray data of *TCD1* in the *Tetrahymena* Functional Gene Database (http://tfgd.ihb.ac.cn)^28^. The conjugation-specific expression patterns suggest that Tcd1 has important functions during the conjugation stage.

### Tcd1 Is Essential for Viable Progeny

**Figure 2:**
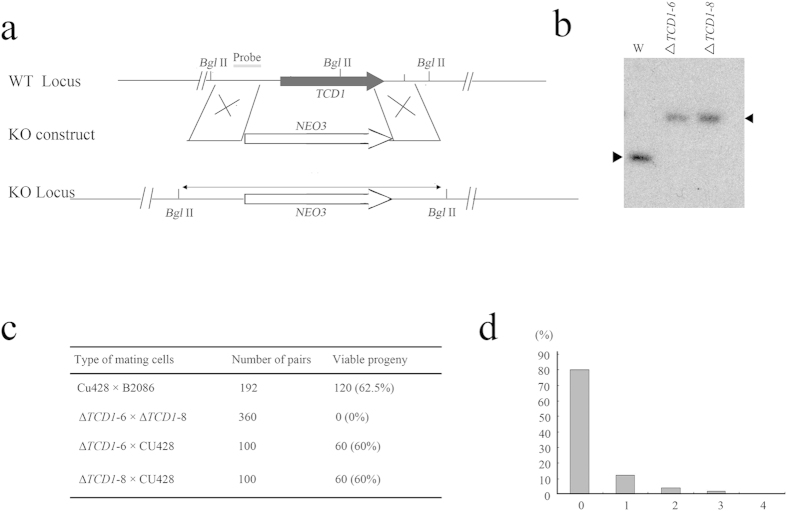
Germline Knockout of *TCD1*. (**a**) Diagram of the *TCD1* knockout. The *neo3* drug resistance marker was inserted into the *TCD1* gene, replacing most of the original coding sequence. (**b**) Southern hybridization of the *TCD1* knockout strains. Total genomic DNA isolated from the knockout strains (Δ*TCD1*-6 and Δ*TCD1*-8) or WT cells was digested with *Bgl* II and hybridized with the *TCD1* probe. Only the correct 3.4 kb band is observed for the knockout strains. (**c**) Viability of progeny. At 12 h to 13 h after mixing, single mating pairs were placed into drops of SPP medium and incubated for 48 h at 30 °C. (d) Division of progeny. Δ*TCD1* cells were mated, and pairs were isolated in drops of SPP medium at 13 h to 14 h after mixing. The number of cells in each drop was counted before the cells died. The results were categorized as follows: 0, no cell division; 1, one division; 2, two cell divisions; 3, three cell divisions; 4, more than four cell divisions.

To investigate the function of Tcd1, we first disrupted the *TCD1* gene in the MAC by homology-directed gene replacement. Similar to wild-type (WT) strains, The *TCD1* somatic knockout strains grew normally during vegetative stage. The development of mating *TCD1* somatic knockout strains is also normal and generated viable progeny during conjugation stage. Then we disrupted the *TCD1* gene in the MAC and MIC. The complete replacement of *TCD1* in the homozygous homokaryon Δ*TCD1*-6 and Δ*TCD1*-8 strains (lacking *TCD1* in both the somatic MAC and the germline MIC) was confirmed by Southern blotting analysis (Fig. 2b). Similar to WT strains, the Δ*TCD1*-6 and Δ*TCD1*-8 strains exhibited normal vegetative growth. Considering that *TCD1* was not expressed during the growth and starvation stages, we speculated that *TCD1* is dispensable at the vegetative stage.

To elucidate the function of Tcd1 during sexual development, the mating progress of Δ*TCD1*-6 and Δ*TCD1*-8 cells was evaluated, and the development stages were analyzed. Although Δ*TCD1* strains mating was delayed and maximum mating was approximately 10% lower than that in WT cells, their sexual development stage was normal and finally stay at the 2MAC-1MIC stage (Supplementary Fig. S1). The Δ*TCD1* mating pairs then were cloned into SPP medium drops and their progeny survival was assessed. The progeny of the Δ*TCD1* cells did not grow upon return to nutrient SPP and eventually died (Figs. 2c,2d). The conjugation-mediated transfer of proteins has been previously reported between two mating cells in *Tetrahymena*^29^. The Δ*TCD1* cells were then mated with WT cells. Progeny viability was rescued when Δ*TCD1* cells were crossed with WT cells (Fig. 2c). The results show that the Δ*TCD1* phenotype was triggered by the absence of *TCD*1. Therefore, Tcd1 is essential for the formation of viable conjugation progeny.

### HA-Tcd1 Can Replace the Function of Endogenous Tcd1

**Figure 3:**
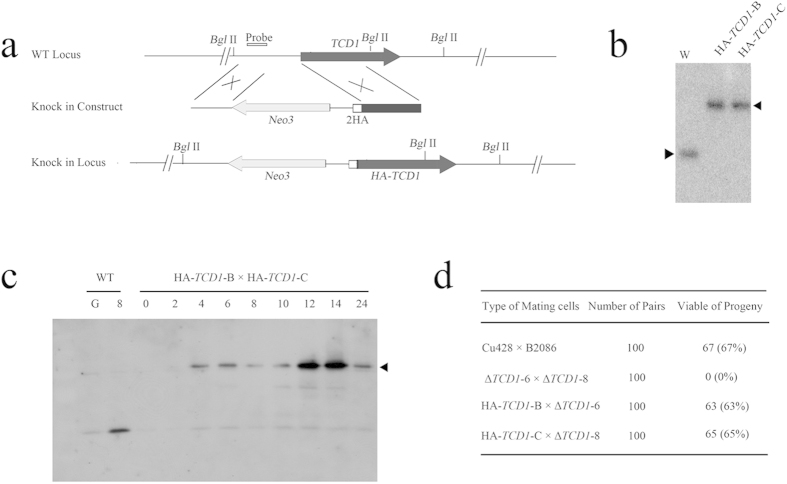
HA2 Tagging of *TCD1*. (**a**) Diagram of the HA2-Tcd1 construct. Two HA epitopes were inserted after the initiation codon of *TCD1.* The *neo3* cassette was inserted into the 5′ nontranscribed sequence. (**b**) Confirmation of complete replacement of endogenous *TCD1* genes by HA-*TCD1*. Total genomic DNA isolated from HA-*TCD1* and WT cells was digested with *Bgl*II and hybridized with the probe shown in (**a**) (**c**) Expression analysis of HA2-Tcd1. Total cell protein was prepared from the mating of HA*-TCD1*-B with HA-*TCD*1-C at 0 h, 2 h, 4 h, 6 h, 8 h, 10 h, 12 h, 14 h, and 24 h after mixing. The extracted protein was separated by SDS-PAGE. HA-Tcd1 was analyzed in Western blots with anti-HA antibody. (**d**) Viability of progeny. Results were obtained using the same method as in Fig. 2c.

A sequence that encodes two HA epitopes was inserted into the 5′ end of the *TCD1* open reading frame to analyze the expression and localization of Tcd1 (Fig. 3a). The cassette Neo3-HA-*TCD1* was introduced into the WT strain CU428 and B2086. Endogenous *TCD1* gene was completely replaced with HA-*TCD1*, as indicated by Southern hybridization (Fig. 3b). The mating progress in HA-tagged *TCD1* cells was normal and similar to that in WT cells. Furthermore, the survival of Δ*TCD1* progeny can be rescued with HA-*TCD1* cells (data not shown). The results indicated that HA-Tcd1 can perform the essential functions of Tcd1. Subsequently, the expression pattern of HA-Tcd1 was examined by Western blot analysis with anti-HA antibodies. A single band of approximately 85 kD was observed, which is consistent with the predicted molecular weight of HA-Tcd1 (Fig. 3c). The expression level of HA-Tcd1 was higher at 12 h to 14 h after mating, which indicates that Tcd1 accumulated at the late conjugation stage. Therefore, Tcd1 could have important functions during the late conjugation stage.

### HA-Tcd1 Dynamically Transfers from the Old MACs to the New MACs

**Figure 4:**
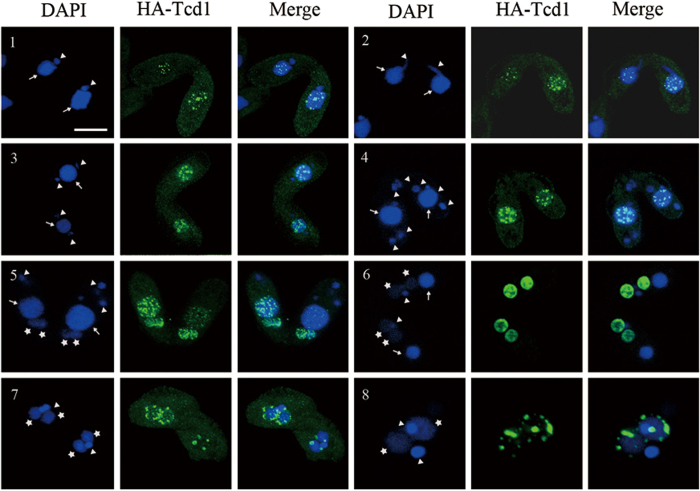
Localization of HA-Tcd1. HA-*TCD1*-B cells and WT cells (CU428) were mated. Cells collected at 3 h, 5 h, 7 h, 9 h, 12 h, and 14 h after mixing were fixed and processed for immunofluorescence staining with anti-HA primary and FITC-conjugated secondary antibodies (middle column). Cells were also stained with DAPI to visualize DNA (left column). Cells underwent pair formation (panel 1), crescent (panel 2), meiosis I (panel 3), meiosis II (panel 4), anlagen (panel 5, 6, 7), pair separation (panel 8). Scale bar, 10 μm.

To understand the function of Tcd1, we analyzed the localization of HA-Tcd1 by anti-HA immunofluorescent staining under the control of its endogenous promoter. HA-Tcd1 was not observed in the MACs and MICs of growing or starved cells. However, HA-Tcd1 was localized from the early to the late conjugation stage. During the conjugation between the HA-*TCD1* and WT strains, HA-Tcd1 initially appeared in only one of the paired cells but was later present in both cells. During the early conjugation stage, HA-Tcd1 was detected in the parental MACs and concentrated in a large number of discrete foci (Fig. 4: panels1-4). Upon the emergence of developing macronuclear anlagen, the staining of the old MACs gradually disappeared, while that of HA-Tcd1 appeared in the developing new MACs. These result indicated that HA-Tcd1 transferred from the old MACs to the developing new MACs (Fig. 4: panel 5). During the early anlagen stage, HA-Tcd1 was evenly distributed throughout the macronuclear anlagen until pair separation (Fig. 4: panel 6). With the differentiation of new MACs into exconjugant, HA-Tcd1 fluorescence became punctuated foci. Subsequently, signal foci gradually decreased and completely disappeared in late exconjugant (Fig. 4: panel 7 and 8). Previous studies reported that Pdd1 is localized to parental MACs, new MACs, and apoptotic MACs while Pdd3 colocalizes with Pdd1 in the peripheral regions of DNA elimination structures^12^,^30^,^31^. Thus, we speculated that HA-Tcd1 can also colocalizes with Pdd1 and display partially functional redundancy.

Aside from macronulear and anlagen localization, transient localization of HA-Tcd1 was observed in the cytoplasm; in addition, the cytoplasmic signal was close to the anterior of the mating pair, that is, the conjusome (Supplementary Fig. S2). The nonmembrane-bound organelles were composed of a coarse reticulum and transiently appeared when developing MACs had initially emerged^32^. Pdd1 is the first component to be found in the conjusome^33^. Given its associating with Pdd1 and IES chromatin, Lia1 is also targeted to this structure^34^. Furthermore, HA-Tcd1 colocalized with Pdd1 in the conjusome (Supplementary Fig. S3). The localization of HA-Tcd1 in the conjusome and its colocalization with Pdd1 in the structure strengthened their connection: these events could be involved in the DNA rearrangement machinery.

### Tcd1 Partially Colocalizes with H3K9me3 in Developing MACs

**Figure 5:**
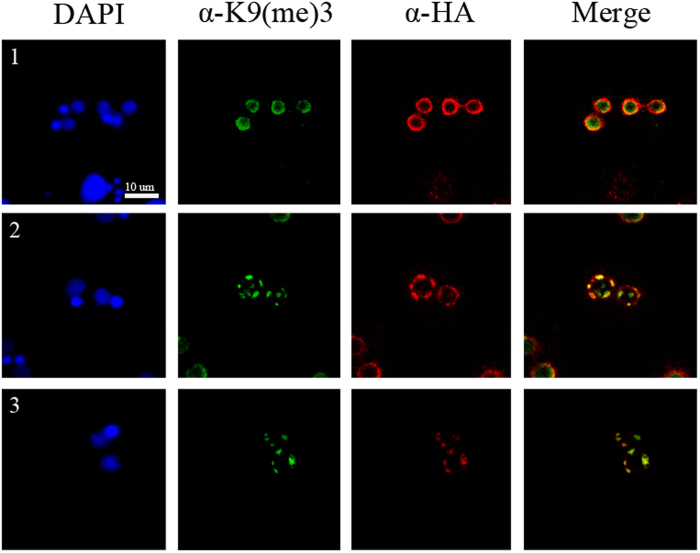
Tcd1 Colocalizes with Trimethylated H3K9 in Developing MACs HA-Tcd1 cells and WT (CU428) were mated and collected at 10 h (**1**), 12 h (**2**) and 14 h (**3**) after mixing. Mating cells were fixed and processed for immunofluorescence with rabbit anti-K9 trimethyl-histone H3 polyclonal antibody and FITC-conjugated secondary antibody (green), or mouse anti-HA monoclonal antibody and Alexa-conjugated secondary antibody (red). Scale bar, 10 μm.

In *Tetrahymena*, H3K9me3 is found only in anlagen and associated with chromatin that contains sequences targeted for elimination^35^. To determine the possible localization of Tcd1 to regions of condensed chromatin in anlagen and its involvement in DNA elimination, we colocalized HA-Tcd1 with H3K9me3 during the anlagen stage. HA-Tcd1 dynamically colocalized with H3K9me3 in most regions during the anlagen stage. Previous studies have shown that H3K9me3 and H3k27me3 are required for heterochromatin formation and IES elimination^12^,^30^,^31^,^36^. Canonical chromodomain of Pdd1p interacts strongly with both H3K27me3 and K9me3, while the chromodomain of Pdd3p preferentially recognizes H3K9me3^12^. In the present study, Tcd1 colocalized with H3K9me3 at the periphery of anlagen (Figs. 5b,5c). Furthermore, the sequence alignment of the canonical chromodomain from Tcd1 and other well characterized readers like HP1, Pdd1, and Pdd3, showed the conserved three aromatic caging residues (Supplementary Fig. S4). The three aromatic residues are predicted to form a “cage” enclosing the methylammonium group of H3K9me3 or/and H3k27me3. The results suggest that Tcd1, similar to Pdd1 and pdd3, could bind to the heterochromatin marker H3K9me3 or H3K27me3 during the late conjugation stage in *Tetrahymena*.

### Tcd1 and Pdd1 Colocalize to the DNA Elimination Structures

**Figure 6:**
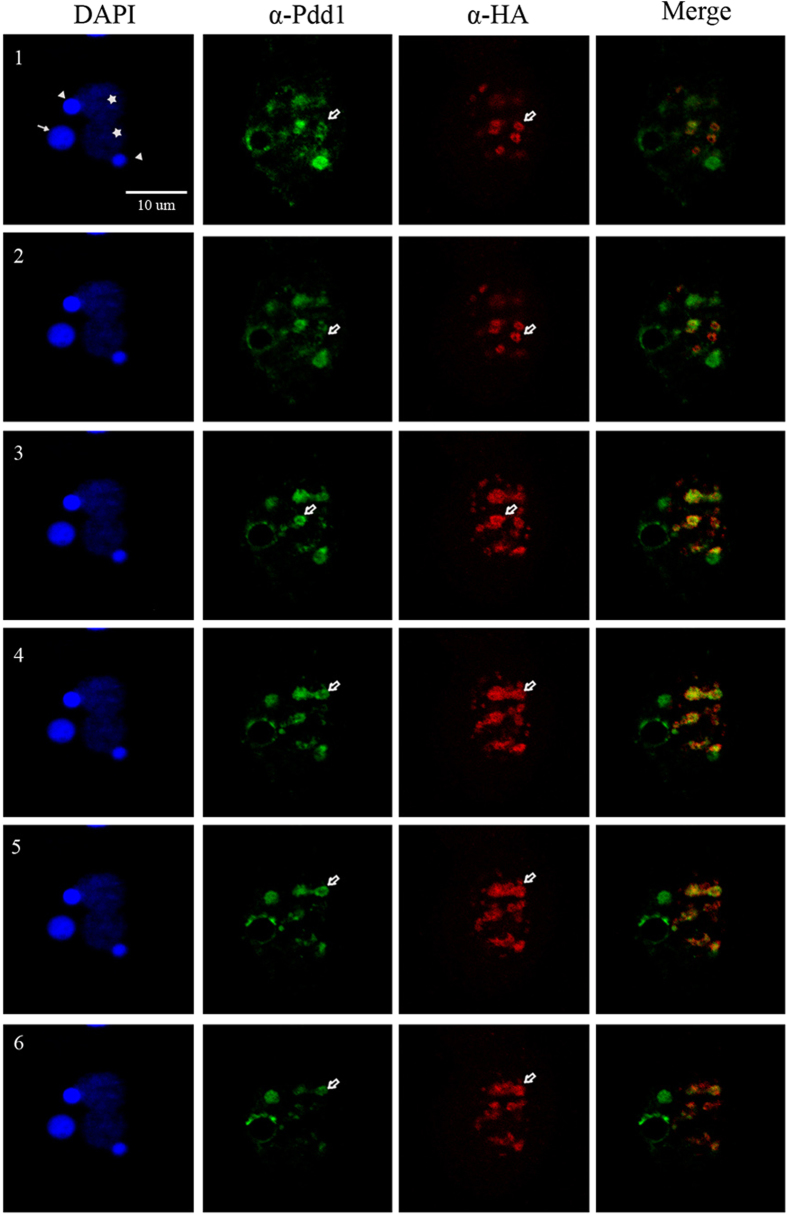
Tcd1 Colocalizes with DNA Elimination Structures Consecutive confocal images (1–6) of a Tetrahymena cell fixed at 12 h and stained with DAPI (column I) and with rabbit anti-Pdd1p primary and FITC-conjugated secondary antibodies (column II) or mouse anti-HA primary and rhodamine-conjugated secondary antibodies (column III). White arrows indicate old MACs, stars indicate anlagen, triangles indicate MICs, and white blank arrows indicate donut-like DNA degradation structures. In most regions, Tcd1 in these conjugations colocalizes with Pdd1 at the donut-like DNA degradation structure (column IV). Scale bar, 10 μm.

Previous data suggested that Pdd1 exists in a complex with eliminated DNA in a characteristic “doughnut-like” structure^31^,^37^. To understand whether or not Tcd1 also occurs in the “doughnut-like” structure, we prepared optical sections at the appropriate anlagen stage via confocal microscopy. Colocalization of Pdd1 and Tcd1 was detected with rabbit anti-Pdd1 and FITC-conjugated secondary antibodies (column II) and mouse anti-HA and rhodamine-conjugated secondary antibodies (column III), respectively. Progressive optical sections demonstrated that Tcd1 and Pdd1 colocalized in the doughnut structure (Fig. 6). A previous study reported that the Pdd1-derived doughnut structure correlates with DNA elimination in *Tetrahymena*^30^. Therefore, Tcd1 could also be involved in DNA elimination.

### DNA Elimination is Defective in Conjugated Δ*TCD1* cells

**Figure 7:**
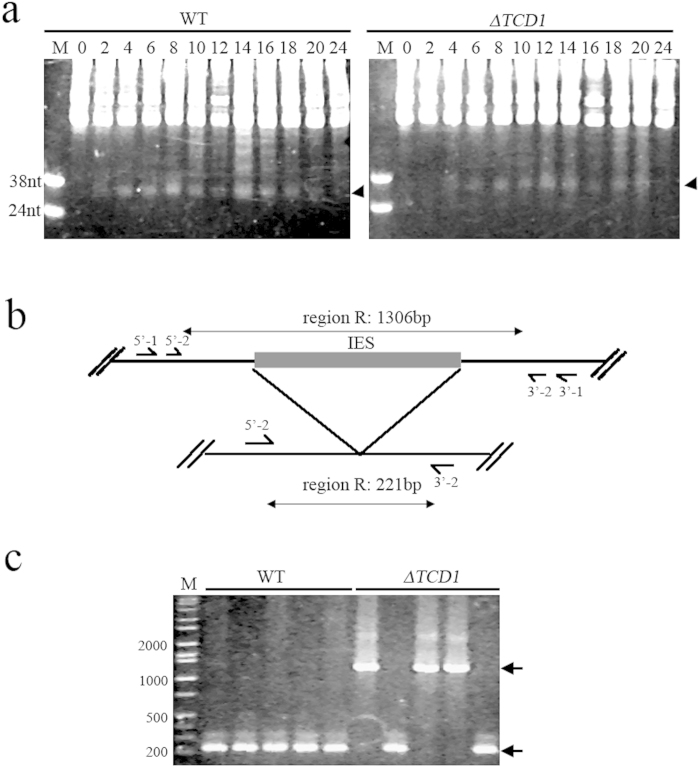
Analysis of Small RNAs and DNA Elimination in the Progeny of *TCD1* Knockout Cells (**a**) Accumulation of small RNA in Δ*TCD1* cells. Total RNA was extracted from mating WT and Δ*TCD1* cells at 0 h, 2 h, 4 h, 6 h, 8 h, 10 h, 12 h, 14 h, 16 h, 18 h, 20 h, and 24 h after mixing. RNA from approximately 5 × 10^4^ cells was fractionated in 12% acrylamide–urea gels and stained by ethidium bromide. Small RNA accumulation and disappearance was unaffected in Δ*TCD1* cells. The position of the small RNA is marked by arrows. DNA oligos (38 and 24 nt) served as markers. (**b**) Schematic of the R element assay. Horizontal lines indicate DNA retained in the macronucleus, and filled box indicates IES. Four primers (arrows on the horizontal lines) were used for nested PCR. The lengths of the expected products are shown at the top and bottom. (**c**) DNA elimination is defective in the conjugated germ line Δ *TCD1* cells. The product sizes of the processed MACs and unprocessed MICs are 221 bp and 1306 bp, respectively, as marked by arrows.

Pdd1 is required for scnRNA accumulation and IES elimination^7^. To understand whether or not Tcd1 affects scnRNA and IES elimination, the total scnRNA was analyzed during life cycle of Δ*TCD1* cells. As shown in Fig. 7a, the scnRNAs generally accumulated and disappeared during the conjugation stage of Δ*TCD1* cells. Subsequently, we used single-cell PCR to analyze the elimination of four IESs (M, R, Cal, and Tlr1 elements) in mating Δ*TCD1* cells. The elimination of M, Cal, and Tlr1 elements in mating Δ*TCD1* cells was similar to that in mating WT cells (data not shown). However, the deletion of R element was aborted in some mating cells (Fig. 7c). Previous results showed that R element is more sensitive to perturbation and more likely to show failed IES deletion than the other DNA deletion elements^4^,^38^. These data imply that Tcd1 is necessary for IES elimination and R element is a sensitive indicator of slight defects during DNA deletion in T*etrahymena*.

### Tcd1 is Required for New Macronuclear Genome Repair

**Figure 8:**
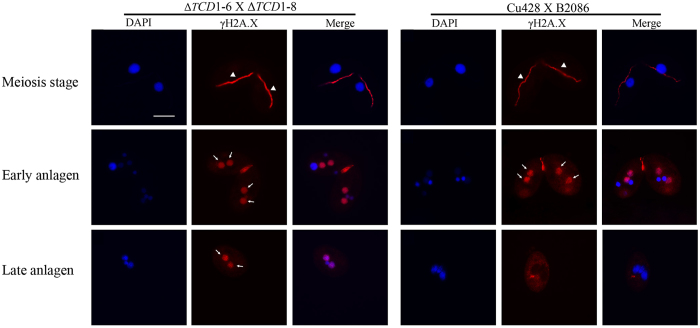
DNA Damage Accumulation in Newly Developed MACs of *TCD1*Knockout Cells. DNA damage accumulation in the new MACs of Δ*TCD1* cells. Immunofluorescence analysis of conjugating cells from mating WT and Δ*TCD1* cells that were stained with anti-γ-H2A.X and DAPI. γ-H2A.X staining occurred at the crescent, early new MACs, and late new MACs of Δ*TCD1* cells, However, no signals were detected in the late new MACs of WT cells. Arrowheads indicate the crescent MICs; arrows indicate the developing MACs. Scale bar, 10 μm.

Partial R element elimination cannot explain the complete failure of development in Δ*TCD1* progeny. Thus, we analyzed DNA repair in the newly developing macronuclear genome. γ-H2A.X signaling is an early cellular response to double-strand breaks. To determine whether or not the *TCD1* knockout induces DNA damage accumulation, we checked the γ-H2A.X signal during *Tetrahymena* conjugation. Fig. 8 shows the immunofluorescence staining of mating WT cells and *TCD*1 knockout cells by using anti-γ-H2A.X antibody. γ-H2A.X staining was observed in mating WT and *TCD1* knockout cells at the early meiotic and early anlagen stages. Surprisingly, γ-H2A.X staining was retained until the late anlagen stage of *TCD1* knockout mating cells. By contrast, the signal disappeared at the late anlagen stage of WT mating cells. The results indicate that Tcd1 is required for genome repair during the development of new macronuclei.

## Discussion

In this paper, we report a novel chromodomain-containing protein (Tcd1) from T*etrahymena*. The following results suggest that Tcd1 is involved in DNA elimination and genome repair. First, *TCD1* expression was undetected in growing or starved cells, but was observed in mating cells at 2 h after mating and increased significantly between 8 h and 12 h when DNA elimination occured; Tcd1 accumulated between 12 h and 14 h when genome repair had finished. Second, Tcd1 was dynamically localized from the parental MACs at the early crescent stage to the new MACs during anlagen formation. Similarly, Tcd1 colocalized with H3K9me3 and Pdd1 in the developing anlagen. Third, Tcd1 was specifically localized in the cytoplasmic conjusome and the nuclear doughnut structure, and interfered with the deletion of R element. Finally, γ-H2A.X staining was delayed to the late anlagen stage in *TCD1* knockout mating cells.

A chromodomain, an evolutionarily conserved domain in a large class of chromatin–binding proteins, performs diverse functions^20^,^39^,^40^. At present, 14 chromodomain-containing proteins have been identified in *Tetrahymena*. Pdd1 is a well-studied member of this family and is required for DNA elimination during new MAC development^41^. Pdd3 containing one chromodomain physically associates with MIC-specific DNA sequences that are targeted for elimination^31^. The chromodomain of Pdd3 preferentially recognizes H3K9me3, the canonical chromodomain of Pdd1 interacts strongly with both H3K27me3 and H3K9me3^12^,^36^. The mutants disrupting H3K27me3 also affected Pdd1 localization^12^. The sequence alignment of the canonical chromodomains from different proteins showed the conserved three aromatic caging residues. The residues are predicted to form a “cage” enclosing the methylammonium group of H3K9me3 or/and H3k27me3. Similar to Pdd1 and Pdd3, Tcd1 also dynamically localized from the parental MACs to the new MACs and colocalized with H3K9me3 at the periphery of anlagen in the present study. Furthermore, Tcd1 and Pdd1 were colocalized in the doughnut structure. The similar staining pattern suggests that Tcd1 is a novel component of the DNA rearrangement machinery with newly evolved functions. The genome of *Saccharomyces pombe* contains at least nine chromodomain-containing proteins (e.g., Swi6, Chp1, Chp2, and Clr4). Swi6 is abundantly expressed and plays a dose-dependent role in forming a repressive structure through its self-association property^45^,^42^. The chromodomain of Chp1 binds to H3K9Me3 and is required for accurate chromosome segregation, whereas that of Chp2 associates with the histone deacetylase complex and is required for histone H3 lysine 14 deacetylation^43^. The *Drosophila* genome possesses at least five HP1 paralogs that have significantly different roles, ranging from the canonical heterochromatic function at the pericentric and telomeric regions to the exclusive localization and regulation of euchromatic genes^44^. In *Tetrahymena*, Pdd1 exhibits dynamic localization, apparently shuttling from the parental to the developing MACs through the conjusome^33^. As a reservoir of components from the old MACs, the conjusome stores or processes them for the newly developing MACs^33^. The recent detection of Lia1, Lia3, and Lia5 in the conjusome further supports the important role of this structure in genome reorganization^32^,^33^,^46^. RNAi components are localized to the chromatoid body in male germ cells. Ago2 is the central protein component of the RNA-induced silencing complex and resides in cytoplasmic processing bodies (P-bodies)^47^. In consideration of the role of small RNAs in directing DNA rearrangement, the conjusome is evolutionarily equivalent to the P-bodies in ciliates^33^. Tcd1 is a novel conjusome component that could be involved in DNA rearrangement directed by an RNAi-related mechanism in *Tetrahymena*.

The development of new MACs in *Tetrahymena* is controlled by an epigenetically regulated, RNAi-related mechanism. Small RNAs are specifically expressed during conjugation do not accumulate in the absence of *TWI1* or *PDD1* expression^7^. By contrast, the global level of scnRNAs was not significantly affected in Δ*TCD1* cells. These results argue that Tcd1 is not directly coupled with the generation and maintenance of scnRNAs in *Tetrahymena*. The protein could be a downstream factor of the process, and its expression profile supports this possibility.

The disruption of *TCD1* did not produce an obvious phenotype during the vegetative growth and starvation stages. The expression of parental *TCD1* is also not essential for sexual development. However, the expression of zygotic *TCD1* is essential to produce viable conjugation progeny. Several protein components (Pdd1, Dcl1, Twi1, Ezl1, and Lia1) involved in IES elimination are required for the development of conjugation progeny^7^,^9^,^12^,^30^,^34^. All the mutant lines that abolished DNA rearrangement failed to complete development, arresting as exconjugants with two new MACs and two new MICs. However, Δ*TCD1* cells completed conjugation and arrested at the exconjugant stage with two new MACs and one new MIC. Once returned to SPP medium, none of the progeny survived. Two possible reasons may explain these phenomena. One is that the elimination of R elements was inhibited in the progeny of Δ*TCD1* strains. The partial deletion phenotype of IES was also previously observed in Δ*EMA1*, Δ*TTN1/TTN2*, and Δ*HEN1* strains^11^,^38^,^48^. The other is that genome repair, an important process in progeny survival, cannot be completed in the developing MACs. The defect in ∆*TKU80* cells involve the rejoining of the IES flanking regions that are retained in the MACs^14^. γ-H2A.X function as a docking site for protein complexes that bind to broken DNA ends and promote chromatin remodeling to join broken chromosomal DNA ends. The chromatin alterations may create structures that favor the recruitment or retention of some DNA repair proteins and thus favor a particular DNA repair pathway. HP1 proteins are involved in reorganization of high-order chromatin structure, which is essential for DNA repair^49^. Moreover, Tcd1 could probably recruit repair factors and remold the chromatin structure to fulfill genome repair at the late conjugation stage in *Tetrahymena*. Future experiments could further determine the different components of the Tcd1 complex and explore the mechanisms by which Tcd1 contributes to chromatin reorganization in genome repair.

## Materials and Methods

### *Tetrahymena* Strains and Culture Conditions

WT strains B2086 (II), CU428 (mpr1-1/mpr1-1 [VII, mp-s]), and CU427 (chx1-1/chx1-1 [VI, cy-s]), as well as micronucleus-defective strains B*VI (VI) and B*VII (VII), were originally obtained from Peter J. Bruns (Cornell University, Ithaca, NY, USA) and are available through the National Tetrahymena Stock Center (http://tetrahymena.vet.cornell.edu/index.html). Cell growth was performed in 1 × SPP medium at 30 °C^24^. Strains were starved overnight in 10 mM Tris-HCl (pH 7.4) prior to mating. Conjugation was induced by mixing populations of complementary mating types at equal cell numbers (2 × 10^5^cells).

### Identification of *TCD1*

*TCD1* (TTHERM_01337400) was identified via a BLAST search of the *Tetrahymena* genome (http://www.ciliate.org) on the basis of its homology with the fission yeast *S. pombe* chromodomain protein Clr4. Additional homologous regions of the Tcd1 protein sequences were identified with Lasergene 7.0 (DNA star). Genomic DNA was prepared from B2086 cells. Total RNA was extracted with Trizol reagent (Invitrogen) from mating B2086 and CU428 cells at 8 h after mixing, the extracted RNA was treated with DNase I (Invitrogen). First-strand cDNA was synthesized using SuperScript III reverse transcriptase (Invitrogen). 3′-RACE and 5′-RACE were performed with the SMART RACE cDNA Amplification Kit (Clontech) to determine the transcription termination and start site, respectively.

### Generation of *TCD1* Knockout Cells and HA-Tagged *TCD1* cells

To generate *TCD1* knockout cells, a 1212 bp fragment of the upstream genomic sequence and a 635 bp fragment of the downstream sequence of *TCD1* were amplified from the genomic DNA of B2086 with the specific primer pairs *TCD1*-5FL-FW/ *TCD1*-5FL-RV and *TCD1*-3FL-FW/*TCD1*-3FL-RV, respectively. A Neo3 cassette was amplified from pBS-MnB-3 with the primer pair Neo3-FW/ Neo3-RV. The knockout construct was created by overlapping PCR with the primer pair *TCD1*-5FL-IFW/ *TCD*1-3-FL-IRV. The *TCD1* flanking sequence in each side of the Neo3 cassette confers paromomycin resistance in *Tetrahymena* cells. The individual constructs were introduced into conjugating CU428 and B2086 cells at 2.5 h by using the Biolistic PDS-1000/He particle-delivery system (Bio-Rad) as previously described^25^. Heterozygous micronuclear transformants were screened by progeny resistance to 100 μg/mL paromomycin and 15 μg/mL 6-methylpurine. Homozygous micronuclear knockout heterokaryons were generated by crossing heterozygous micronuclear transformants with B*VI or B*VII star strains. Homozygous micronuclear knockout heterokaryons were identified by paromomycin/CdCl_2_ sensitivity and verified through crosses with CU427, which produced progeny resistant to 100 μg/mL paromomycin and 25 μg/mL cycloheximide. Homozygous homokaryon *TCD1* knockout cells (Δ*TCD1*-6 and Δ*TCD*1-8) were generated by crossing homozygous heterokaryons of compatible mating types^26^. 10 μg DNA was digested with *Bgl*II, and the resulting fragments were separated in agarose gel followed by blotting onto a nylon membrane. The membrane was probed with the labeled short PCR product of the *TCD1* 5′ N-terminal fragment for Southern blot analysis. The localization of the probes was visualized by autoradiography.

To generate HA tagged *TCD1* cells, the 5′ and 3′ fragments of HA-*TCD1* were amplified from genomic DNA with the primer pairs *TCD1-*HA2-FW1/*TCD1*-NHA-RV and *TCD1*-NHA-FW/*TCD1*-HA2-RV1, respectively. The full HA-*TCD*1 sequence was obtained from the 5′ and 3′ fragment of HA-*TCD*1 via overlapping PCR with the primer pair *TCD1*-HA2-in-FW2/*TCD1*-HA2-in-RV2. The Neo3 cassette was amplified from pBS-MnB-3 with the prime pair *TCD1*-NEO-5FW/ *TCD1*-NEO-3RV. The over-lapping amplified product was cloned into a plasmid vector, and the Neo3 cassette was introduced into the *Hpa I* site in the 5′-nontranscribed sequence of *TCD*1. The constructs were introduced into CU428 and B2086 cells via the Biolistic PDS-1000/He particle-delivery system. The endogenous *TCD1* was completely replaced with HA-*TCD1* by phenotypic assortment and selection in increasing concentrations of paromomycin. Complete replacement was confirmed by Southern hybridization.

### Western Blot Analysis

Whole-cell proteins from approximately 2.5 × 10^3^ cells were separated by 12.5% sodium dodecyl sulfate-polyacrylamide gel electrophoresis and then transferred onto PVDF membranes. Blots were incubated with 1: 2, 000 dilution of mouse anti-HA antibody (Covance, Berkeley, CA, USA) in blocking solution (5% milk, 0.1% Tween 20 in PBS) and visualized by incubation with a 1: 5, 000 dilution of HRP-conjugated anti-mouse IgG antibody (Zymed Labs Inc., South San Francisco, CA, USA) in blocking solution. Finally, the blots were reacted with Western Blot Chemiluminescence Reagent (NEN Life Science, Boston, MA, USA).

### Immunofluorescence Staining

Cells were fixed in Lavdowsky’s fixative (ethanol: formalin: acetic acid: water = 50: 10: 1: 39) or partial Shaudin’s fixative overnight at 4 °C and then immobilized on cover glasses coated poly-L-lysine (Sigma)^7^,^27^. Cells were processed with a 1: 2, 000 dilution of anti-HA antiserum, a 1: 500 dilution of anti-pdd1 serum (provided by Dr. C. David Allis, The Rockefeller University, New York, NY, USA), a 1: 200 dilution of anti-trimethyl histone Lys-9 antibody (Upstate), or a 1: 100 dilition of anti-γ-H2A.X antibody (Millipore) and then incubated in a 1: 2, 000 dilution of Alexa488-conjugated anti-rabbit IgG (Invitrogen) or a 1: 200 dilution of FITC-conjugated anti-rabbit IgG antibody (Zymed Labs Inc.). The samples were incubated with 1 μg/mL 4′, 6-diamidine-2-phenylindole dihydrochloride (Roche) in PBS. Digital images were captured with an Olympus BH-2 fluorescence microscope or a Leica TCS SP confocal microscope and processed using Adobe Photoshop.

### Analysis of Small RNA

Total RNA was extracted with TRIzol reagent, separated on 12% polyacrylamide-urea DNA sequencing gels, and then visualized as previously described^7^.

### Analysis of IES Processing

Mating pairs were cloned into drops of 10 mM Tris-HCl (pH 7.4) at 12 h after mixing and then incubated for 24 h at 30 °C. One of the separated exconjugants was transferred to 1 μL lysis buffer, incubated at 37 °C for 30 min, and then boiled for 3 min. The cell lysate was used for the first set of PCR (40 cycles) at a final volume of 25 μL, and 1 μL of the first reaction was used for the second set of PCR (40 cycles). The following primers were used: R5-1/R5-2; R3-1/R3-2^7^.

## Author Contributions

J.X., A.L. and W.W. conceived and coordinated the experiments. J.X., W.W. and Y.Y. performed the experiments. J.X. and W.W. wrote the manuscript. All authors reviewed the manuscript.

## Additional Information

**How to cite this article**: Xu, J. *et al*. Chromodomain protein Tcd1 is required for macronuclear genome rearrangement and repair in *Tetrahymena*. *Sci. Rep.* **5**, 10243; doi: 10.1038/srep10243 (2015).

## Supplementary Material

Supplementary Information
