# Supplementary material for: Chromodomain protein Tcd1 is required for macronuclear genome rearrangement and repair in Tetrahymena
Source: Sci Rep. 2015 May 19;5:10243. doi: 10.1038/srep10243 (PMC4437310; doi:10.1038/srep10243)
Supplement: Supplementary Information [file srep10243-s1.doc]

Supplementary Information

**Chromodomain protein Tcd1 is required for macronuclear genome rearrangement and repair in *Tetrahymena***

Jing Xu^1,2^, Yajing Yuan^2^, Aihua Liang^2^, Wei Wang^2^,*

1. College of Life Science, Shanxi University, Taiyuan, 030006, China

2. Key Laboratory of Chemical Biology and Molecular Engineering of Ministry of Education, Institute of Biotechnology, Shanxi University, Taiyuan, 030006, China

*Correspondence: gene@sxu.edu.cn

Phone: 86-351-7011499

Fax: 86-351-7011499

**Figure S1. Developmental Profiles of WT and *TCD1* Knockout cells during Conjugation.**

Starved WT cells (B2086×CU428) and *TCD*1 knockout strains (Δ*TCD1-*6×Δ*TCD1-*8) were mixed. The phenotypes were observed by DAPI staining at 2 h, 4 h, 6 h, 8 h, 10 h, 12 h, 14 h, 16 h, 24 h, and 36 h after mixing. The stages were categorized with columns of different colors: blue, single cell; dark red, crescent; yellow, meiosis; cyan, nuclear selection; brown, macronuclear anlagen; peach, nuclear alignment; navy blue, pair separation; teal blue, one MIC and two MACs.

**Figure S2. HA-Tcd1 Localizes in the Conjusome during Anlagen Formation**

Cells were fixed and processed for immunofluorescence staining with mouse anti-HA primary and FITC conjugated secondary antibodies (middle column). Cells were also stained with DAPI (left column). Black arrows indicate the MACs, arrowheads indicate the MICs, Stars indicate anlagen, blank arrows indicate the conjusome. Scale bar, 10 µm.

**Figure S3. Colocalization of Tcd1 and Pdd1 in the Conjusome**

Cells were fixed and processed for immunofluorescence staining with rabbit anti-Pdd1 and FITC-conjugated secondary antibodies (column II) and mouse anti-HA primary and rhodamine conjugated secondary antibodies (column III). Cells were also stained with DAPI (left column). Blank arrows indicate the conjusome, stars indicate new MACs; arrows indicate old MACs, arrowheads incidate MICs. Scale bar, 10µm.

**Figure S4. Alignment of Canonical Chromodomain of Tcd1 with the Chromodomain of other proteins**

Alignment of canonical chromodomain of Tcd1 with the chromodomain of other proteins. *Tetrahymena thermophila* Pdd1(AAB61684), *Tetrahymena thermophila* Pdd3(AAF36692), *Tetrahymena thermophila* Tcd1(EAR82061), T*etrahymena thermophila* Hhp1(AAC78328), *Drosophila melanogaster* HP1(AAF52618), *Drosophila melanogaster* Pc(AAL49241), *Schizosaccharomyces pombe* Clr4(AAC18302). The three aromatic residues (shown in asterisk) are predicted to form a “cage” enclosing the methylammonium group of H3K9me3 and H3k27me3.

**Figure S1. Developmental Profiles of WT and *TCD1* Knockout cells during Conjugation.**

**
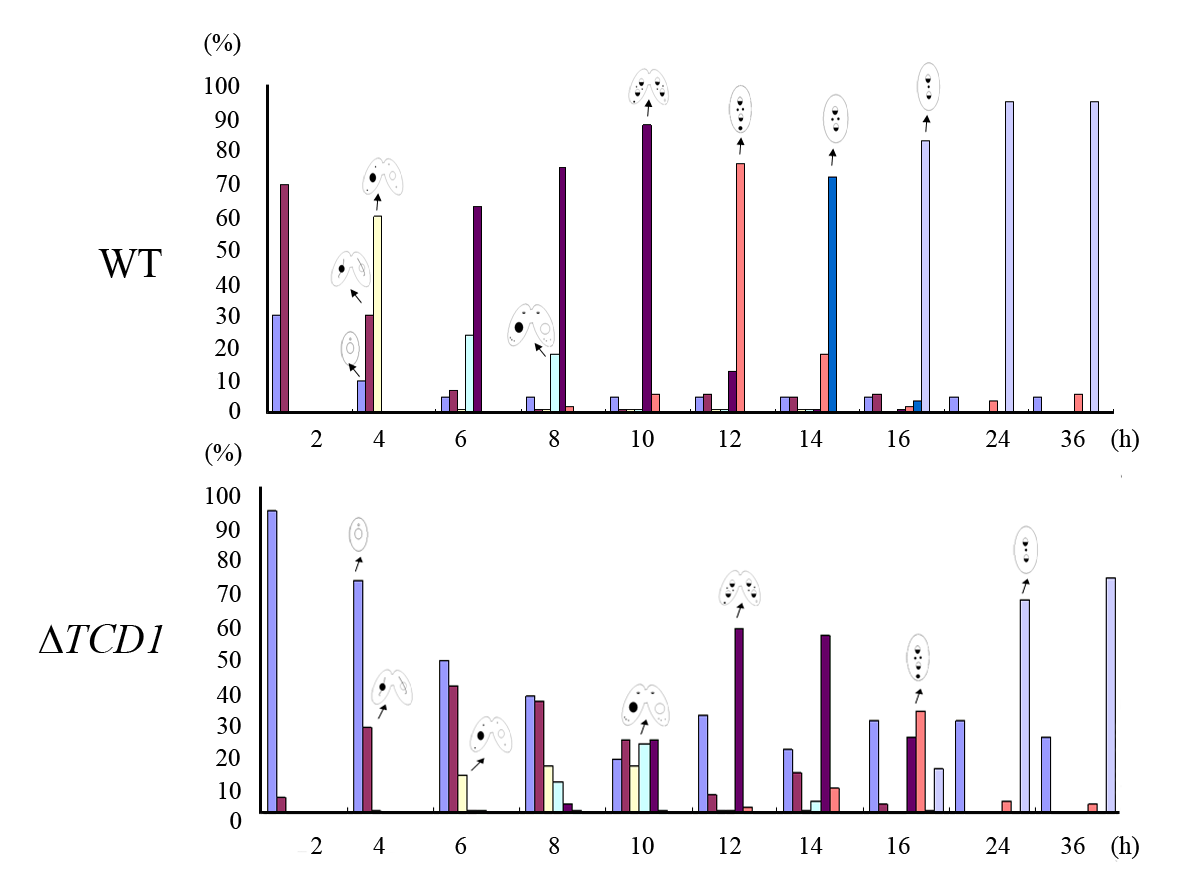
**

**Figure S2. HA-Tcd1 Localizes in the Conjusome during Anlagen Formation**


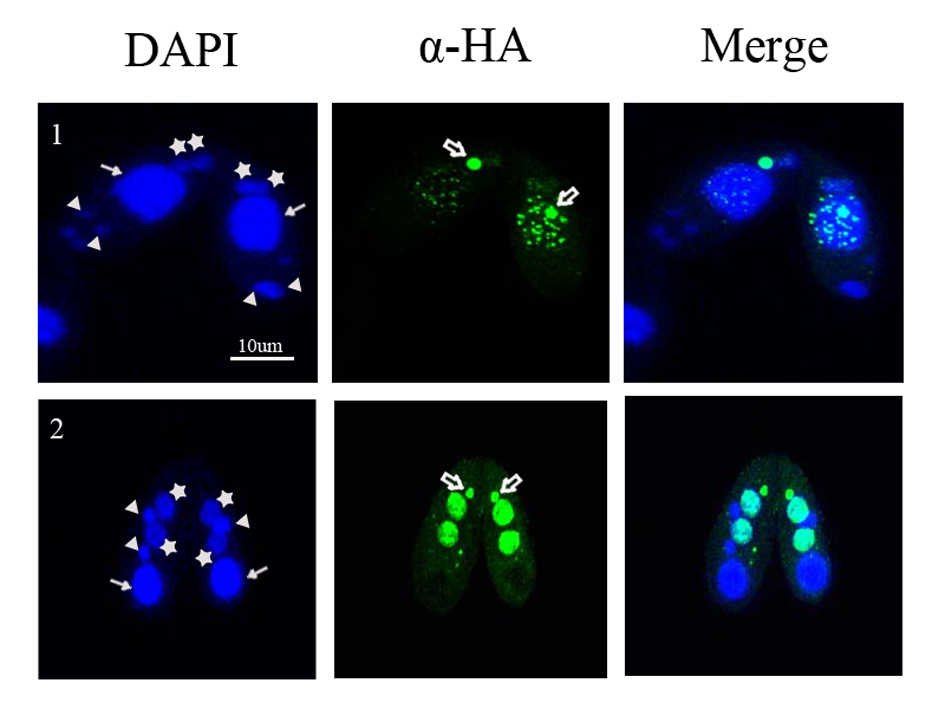


**Figure S3. Colocalization of Tcd1 and Pdd1 in the Conjusome**


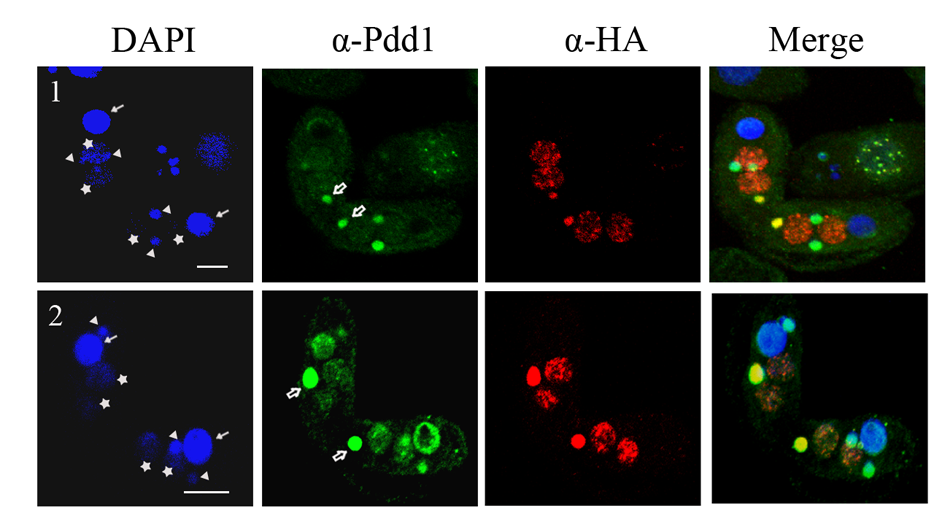


**Figure S4. Alignment of Canonical Chromodomain of Tcd1 with the Chromodomain of other proteins**

**
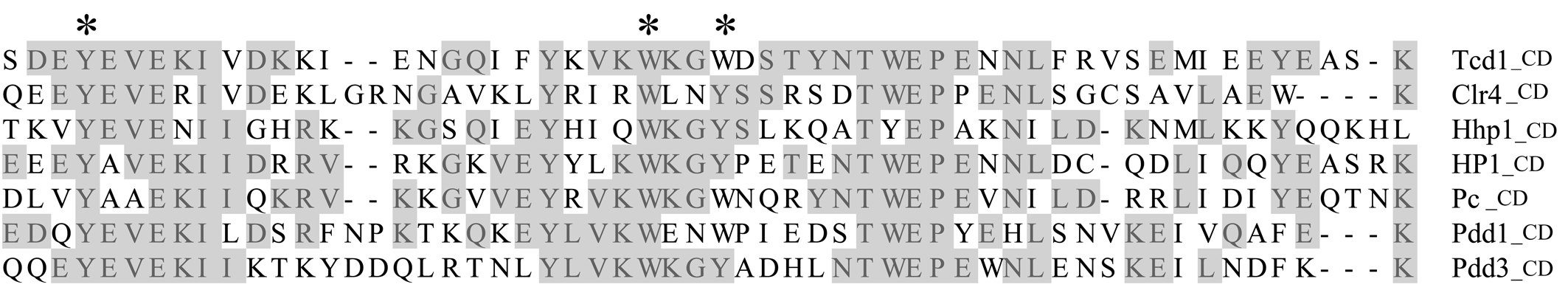
**

**Table S1. Primers Used in This Study**

| Primer Names | Sequences (5′ to 3′) |
| --- | --- |
| *TCD1* cDNA cloning |  |
| *TCD1*-FW | ATGTTCACTGTAAAGCAACAGGCAC |
| *TCD1*-RV | TCATTTAGCTATTTATTACTATTATTTC |
| *TCD1*-5-ORp | CAAAGCTGAACCACCGTTCCCTTAGT |
| *TCD1*-5-IRp | GCCTCTAGACAGCTTATCCTTTGATGCAAGAAACT |
| *TCD1*-3-OFp | GAACCTTGTGACTAATCTACTCTACA |
| *TCD1*-IFp | GCGTCTAGACAAGAGTTAATGGTTGTGTACCTCT |
| *TCD1* knockout cassette |  |
| *TCD1*-5FL-FW | TGAAGAGATCTTGAATACAAAGCTTGGAT |
| *TCD1*-5FL-RV | *GTGTATTTAAATTAAAGGAGTTATTCA*AGTTTACGAGTTTAGCTAATATAAACA |
| *TCD1*-3FL-FW | *CAAATTTTTACTGGAAAAATGC*TTGAAGAGGTTTAATAAGACAAAGA |
| *TCD1*-3FL-RV | AGCCTACTGGATCATGTTCTTTTAGCAT |
| *TCD1*-5FL-IFW | CTGTGTATAAGAAAGCAATGATTTCAT |
| *TCD*1-3-FL-IRV | ACTTTGAAAGTAGTCTTAGAAAGATCT |
| Neo3-FW | GAATAACTCCTTTAATTTAAATACAC |
| Neo3-RV | GCATTTTTCCAGTAAAAATTTGA |
| HA-*TCD1* knockin cassette |  |
| *TCD1-*HA2-FW1 | AGACAAATTAAGCGCTTCATGTTTGCT |
| *TCD1*-HA2-in-FW2 | TAAG*GGTACC*TGTGCTTTTCCAGTAATAAACAAAC |
| *TCD1*-HA2-RV1 | GCAGAAGTAGAAGAAGCAGATTATGA |
| *TCD1*-HA2-in-RV2 | TAAC*GAGCTC*CATATTTTAATTCTTTTCGTATGTATCC |
| *TCD1*-NHA-FW | CCTGATTATGCTTATCCATATGATGTTCCTGATTATGCTTTCACTGTAAAGCAACAGGCACTAA |
| *TCD1*-NHA-RV | CATATGGATAAGCATAATCAGGAACATCATAAGGATACATCTACACAAAAGGTTTTTTTTAA |
| *TCD1*-NEO-5FW | CTAG*GTTAAC*GAATAACTCCTTTAATTTAAATACAC |
| *TCD1*-NEO-3RV | TCAC*GTTAAC*GCATTTTTCCAGTAAAAATTTGA |
